# Comprehensive RNA sequencing in primary murine keratinocytes and fibroblasts identifies novel biomarkers and provides potential therapeutic targets for skin-related diseases

**DOI:** 10.1186/s11658-021-00285-6

**Published:** 2021-10-03

**Authors:** Tiancheng Wang, Zhenwei Zhou, Enjing Luo, Jinghong Zhong, Daqing Zhao, Haisi Dong, Baojin Yao

**Affiliations:** Jilin Ginseng Academy, Changchun University of Chinese Medicine, Changchun, 130117 China

**Keywords:** Skin, Keratinocytes, Fibroblasts, RNA-seq, Biomarkers, Potential therapeutic targets

## Abstract

**Background:**

Keratinocytes and fibroblasts represent the major cell types in the epidermis and dermis of the skin and play a significant role in maintenance of skin homeostasis. However, the biological characteristics of keratinocytes and fibroblasts remain to be elucidated. The purpose of this study was to compare the gene expression pattern between keratinocytes and fibroblasts and to explore novel biomarker genes so as to provide potential therapeutic targets for skin-related diseases such as burns, wounds, and aging.

**Methods:**

Skin keratinocytes and fibroblasts were isolated from newborn mice. To fully understand the heterogeneity of gene expression between keratinocytes and fibroblasts, differentially expressed genes (DEGs) between the two cell types were detected by RNA-seq technology. Quantitative real-time polymerase chain reaction (qRT-PCR) was used to detect the known genes of keratinocytes and fibroblasts and verify the RNA-seq results.

**Results:**

Transcriptomic data showed a total of 4309 DEGs (fold-change > 1.5 and q-value < 0.05). Among them, 2197 genes were highly expressed in fibroblasts and included 10 genes encoding collagen, 16 genes encoding transcription factors, and 14 genes encoding growth factors. Simultaneously, 2112 genes were highly expressed in keratinocytes and included 7 genes encoding collagen, 14 genes encoding transcription factors, and 8 genes encoding growth factors. Furthermore, we summarized 279 genes specifically expressed in keratinocytes and 33 genes specifically expressed in fibroblasts, which may represent distinct molecular signatures of each cell type. Additionally, we observed some novel specific biomarkers for fibroblasts such as Plac8 (placenta-specific 8), Agtr2 (angiotensin II receptor, type 2), Serping1 (serpin peptidase inhibitor, clade G, member 1), Ly6c1 (lymphocyte antigen 6 complex, locus C1), Dpt (dermatopontin), and some novel specific biomarkers for keratinocytes such as Ly6a (lymphocyte antigen 6 complex, locus A) and Lce3c (late cornified envelope 3C), Ccer2 (coiled-coil glutamate-rich protein 2), Col18a1 (collagen, type XVIII, alpha 1) and Col17a1 (collagen type XVII, alpha 1). In summary, these data provided novel identifying biomarkers for two cell types, which can provide a resource of DEGs for further investigations.

**Supplementary Information:**

The online version contains supplementary material available at 10.1186/s11658-021-00285-6.

## Background

Skin is the biggest organ of the human body. Its functions include barrier, immunity, gender communication, body temperature regulation, and wound healing. The skin is usually divided into three layers: epidermis, dermis, and subcutaneous tissue [1–3]. The epidermis is mainly composed of keratinocytes. It provides a protective barrier from the external environment such as UVB irradiation and pathogens [4]. The dermis, located between the epidermis and subcutaneous tissue, is mainly composed of fibroblasts and rich in collagen. It provides structural support for the skin [5]. The functions of fibroblasts include the synthesis of collagen and elastin, secretion of matrix fluid, and degradation of fibrous and nonfibrous connective tissue matrix proteins [6]. Collagen and elastin are fundamental to maintaining healthy skin, and their reduction can result in skin roughness and wrinkles, subsequently causing skin senescence. Therefore, strategies that increase fibroblast proliferation and expression of related genes or reduce fibroblast apoptosis may be effective for addressing the issue of senescence.

Skin diseases, including skin injuries, senescence, and scars, pose challenges for patients and physicians. Although some treatments are currently available, the precise restoration of these skin defects remains a huge challenge for dermatologists due to insufficient knowledge about the skin-related cells. Exploring the differential gene expression in fibroblasts and keratinocytes is helpful to analyze the important gene networks. We can also determine the expression levels and location of these genes (epidermis and dermis). For example, keratin is mainly related to the skin barrier and water balance in the epidermis, and type I collagen and fibronectin are mainly in fibroblasts, which are related to wound healing. Thus, this may provide a therapeutic target for precision medicine in skin-related diseases. In addition, fibroblast cell therapy has been used to treat burns, diabetic wounds, scars, and aging skin. Therefore, acquiring the gene expression profiles of fibroblasts is important for treating skin-related diseases by better understanding and separation of fibroblasts. Although it has been reported that the gene expression patterns between keratinocytes and fibroblasts can be compared using cDNA microarray analysis, the microarray platform only profiles the predefined transcripts through hybridization, and it does not allow for full sequencing of the whole transcriptome [7]. With advances in next-generation sequencing technologies, it is possible to obtain genome-wide high-throughput data with high-throughput RNA sequencing (RNA-seq) technology. RNA-seq technology is a powerful and efficient method for transcriptome analysis with higher coverage and greater sensitivity. The wide use of RNA-seq enables a better understanding of comprehensive transcriptomic information such as alternative splicing, noncoding RNAs, mRNA, and small RNA. A major application of this technology is the sequencing of mRNA expression; thus, it has become a useful tool in many whole-cell transcriptome studies [8–10]. Herein, we report the transcriptome of skin keratinocytes and fibroblasts using RNA-seq. We analyzed and compared the expression profiles of RNA-seq data between skin keratinocytes and fibroblasts to identify DEGs and define specific type of cells. To validate our dataset, we randomly selected and analyzed the expression of 10 genes, including Lce3c, Ccer2 (coiled-coil glutamate-rich protein 2), Cnfn (cornifelin), Sprr2h (small proline-rich protein 2H), Plac9a (placenta-specific9a), Ccl12 (chemokine (C–C motif) ligand 12), Car3 (carbonic anhydrase 3), and Plac8 (placenta-specific 8), using qRT-PCR. Our data not only provide an important insight for the specification of both skin keratinocytes and fibroblasts but also constitute a proven resource for further research on the treatment of skin disorders such as burns, ulcers, and wounds.

## Methods

### Keratinocyte and fibroblast isolation and culture

Skin samples were taken from a total of eight newborn male mice, and keratinocytes and fibroblasts were isolated in accordance with the previously reported method [11]. In brief, eight mice were euthanized and their skin was separated and placed in sterile dishes on ice. The skin tissues were then floated on 0.25% trypsin without EDTA (Invitrogen, USA) at 4 °C overnight. The next day, the skin tissues were washed in phosphate-buffered saline (PBS) and divided into the dermis and epidermis using fine tweezers. The epidermis was transferred to keratinocyte serum-free medium (K-SFM, Gibco, USA) and minced with scissors. Subsequently, tissue fragments were incubated with 1 mg/mL collagenase II at 37 °C for 1 h. Then, cells were collected and centrifugally washed at 400*g* for 5 min with K-SFM, and finally cultured in K-SFM, which has a Ca^2+^ concentration less than 0.1 mM [11, 12]. When keratinocytes reached 80% to 90% confluence, cells were collected as follows. First, cell culture medium was removed and then 0.25% trypsin was added to digest cells for 10 min at 37 °C. Finally, complete medium was added to inactivate trypsin and cells were collected by centrifugation (1100 rpm/5 min). The dermis was digested with 3 mg/mL collagenase II (Sigma, USA) for 45 min at 37 °C on a shaker. After that, Dulbecco’s Modified Eagle Medium (DMEM, Gibco, USA) supplemented with 10% FBS (Fetal Bovine Serum, Gibco, USA) was added to stop the digestion. The cells were collected and centrifuged at 150*g* for 5 min and then cultured in DMEM supplemented with 10% FBS at 37 °C in 5% CO_2_. When cells reached 80% to 90% confluence, they were sub-cultured on plates as follows: First, cell supernatant was taken out, then 0.25% trypsin was added to digest cells at 37 °C for 4 min. Finally, cells were put into the incubator and cultured continuously. Fibroblasts and keratinocytes were passaged once and used in experiments.

### Purity analysis of primary keratinocytes and fibroblasts

To analyze the purity of keratinocytes and fibroblasts, cells were incubated with 4% paraformaldehyde for 30 min, and then added to 0.1% Triton for 20 min at room temperature. Subsequently, cells were incubated with primary antibodies (rabbit anti-FSP1/S100a4, rabbit anti-KRT5, Abcam, USA) at room temperature for 30 min. Then Alex-conjugated antibodies (Abcam, USA) were added and incubated for 30 min. After washing with PBS-T (PBS containing 0.2% Tween-20), cells were resuspended in PBS. For flow cytometric analysis, 5000 cells were counted and analyzed by a FlowSight Imaging Flow Cytometer (Merck Millipore, USA). Analysis of cell purity was performed using the IDEAS Application V6.1 software (Amnis, USA) [13].

### RNA extraction and cDNA library preparation

Total RNA was extracted from keratinocytes and fibroblasts using TRIzol (Tiangen, Beijing, China) in line with the manufacturer’s instructions. The quality of RNA samples was evaluated by agarose gel electrophoresis and the Bioanalyzer 2100 system (Agilent Technologies, USA). Paired-end cDNA libraries were generated using a TruSeq Stranded mRNA kit (Illumina, USA) in accordance with the manufacturer’s protocol. Briefly, Dynal oligo(dT) beads were used to separate poly(A) mRNA from the total RNA samples, and then the mRNA was fragmented into short fragments of ~ 200 bp by fragment buffer treatment, followed by reverse transcription to first-strand cDNA by random primers. The second-strand cDNA was synthesized by adding buffer, dNTPs, RNaseH, and DNA polymerase I. After that, the double-stranded cDNA was purified and end-repaired to add ‘a-tail’ for Illumina adaptor ligation. Finally, the integrity of cDNA was evaluated by the Bioanalyzer 2100 system (Agilent Technologies, USA) again, and then cDNAs were subjected to RNA sequencing [14, 15].

### RNA sequencing and data analysis

After construction of cDNA libraries, RNA sequencing was performed on an Illumina HiSeq 2500 platform (Illumina, USA). The resulting sequences were filtered to obtain high-quality sequences, clean reads, and then uploaded in the NCBI Sequence Read Archive (SRA) database (Accession Number: PRJNA589753). Subsequently, the clean reads were aligned with the mouse reference genome sequence using Hierarchical Indexing for Spliced Alignment of Transcripts (HISAT). The NR, KEGG (Kyoto Encyclopedia of Genes and Genomes), and GO databases were used to perform gene annotation and functional assignments. The fragment per kb per million reads (FPKM) method was used to evaluate the gene expression level. DESeq2 was utilized to detect differentially expressed genes (DEGs), and the genes with a log_2_ fold-change ≥ 1 or ≤  − 1 and with a p value ≤ 0.05 were selected.

### Real-time quantitative PCR analysis

Total RNA was extracted from keratinocytes and fibroblasts using TRIzol (Tiangen, China) in line with the manufacturer’s instructions [16]. First-strand cDNA was synthesized with the PrimeScript RT reagent kit (TaKaRa, Japan) and used for qRT-PCR reactions performed on a Bio-Rad instrument (Bio-Rad, USA) with TB Green Premix Ex Taq II (TaKaRa, Japan). The relative mRNA expression levels of the target genes were expressed as 2^−ΔΔCT^, and the Gapdh gene served as an internal reference to normalize the target gene expression levels. The primers were designed as shown in Additional file 1: Table S1 and Additional file 8: Table S8.

### Statistical analyses

QRT-PCR data were expressed as the mean ± standard deviation (SD) from at least three independent experiments and analyzed in Excel. The difference between the two groups was determined using the unpaired t test. A p value < 0.05 was considered statistically significant.

## Results

### Identification of keratinocytes and fibroblasts by qRT-PCR

**Fig. 1:**
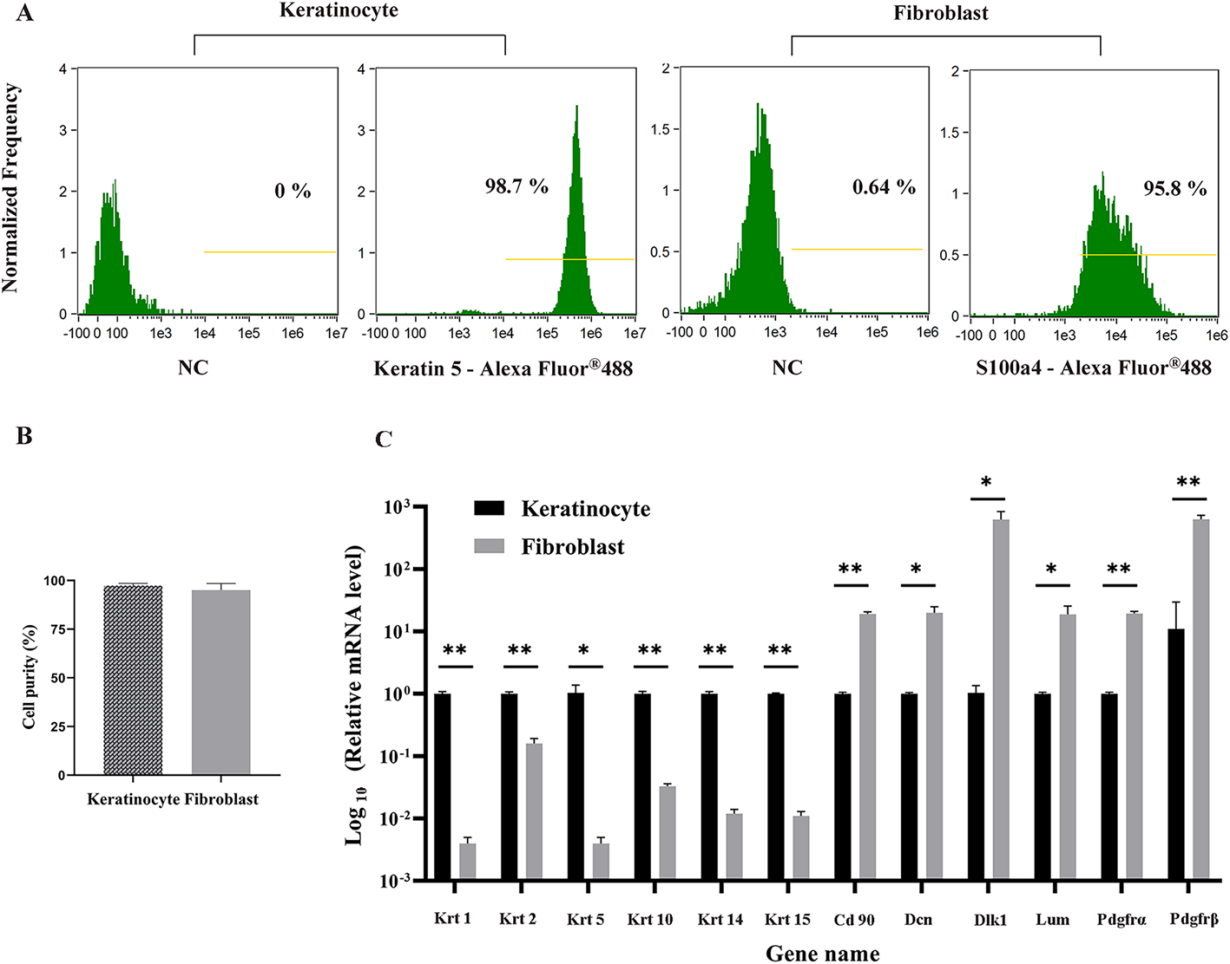
Identification of keratinocytes and fibroblasts. **A** The purity of primary keratinocytes and fibroblasts was identified by flow cytometry. **B** The relative gene expression levels of known marker genes of keratinocytes and fibroblasts were detected by qRT-PCR. The relative mRNA expression was expressed as 2^−ΔΔCT^, the Gapdh gene served as an internal reference to normalize the target gene expressions. Data are presented as the mean ± SD from at least three independent experiments. *P < 0.05, **P < 0.01

Flow cytometry was performed to analyze the purity of these cells. As shown in Fig. 1A, the purity of keratinocytes was 98.7% and that of fibroblasts was 95.8%. The cells without treatment were used as a negative control. To further characterize the isolated cells, multiplex qRT-PCR was performed on 12 known genes—Krt1 (keratin 1), Krt2 (keratin 2), Krt5 (keratin 5), Krt10 (keratin 10), Krt14 (keratin 14), KRT15 (keratin 15), CD90 (thymus cell antigen 1, theta), Dcn (decorin), Dlk1 (delta-like 1 homolog Drosophila), Lum (lumican), Pdgfrα (platelet-derived growth factor receptor, alpha polypeptide), and Pdgfrβ (platelet-derived growth factor receptor, beta polypeptide)—that had previously been reported as markers to distinguish keratinocytes from fibroblasts [5, 8, 17–20]. The relative fold changes of each gene in the qRT-PCR results are shown in Fig. 1B. As expected, Krt1, Krt2, Krt5, Krt10, Krt14, and Krt15 were highly expressed in keratinocytes, and Cd90, Dcn, Dlk1, Lum, Pdgfrα, and Pdgfrβ were highly expressed in fibroblasts. Therefore, the results confirmed the successful isolation of keratinocytes and fibroblasts from the mice skin tissues.

### Characterization of transcriptome sequencing data

In this study, two samples were tested by sequencing on the Illumina HiSeq platform; we obtained about 7.34 Gb of data for each sample. As shown in Additional file 2: Table S2, after restrictive filtering of the raw data, an aggregate of 47,468,704 and 50,373,090 clean reads were obtained from keratinocytes and fibroblasts, respectively, and an average of 94% of reads were mapped to the mouse reference genome. The quality analysis of the clean reads suggested that the Q30 quality score was over 93%, and the GC percentage was approximately 52%.

### Identification of DEGs

**Fig. 2:**
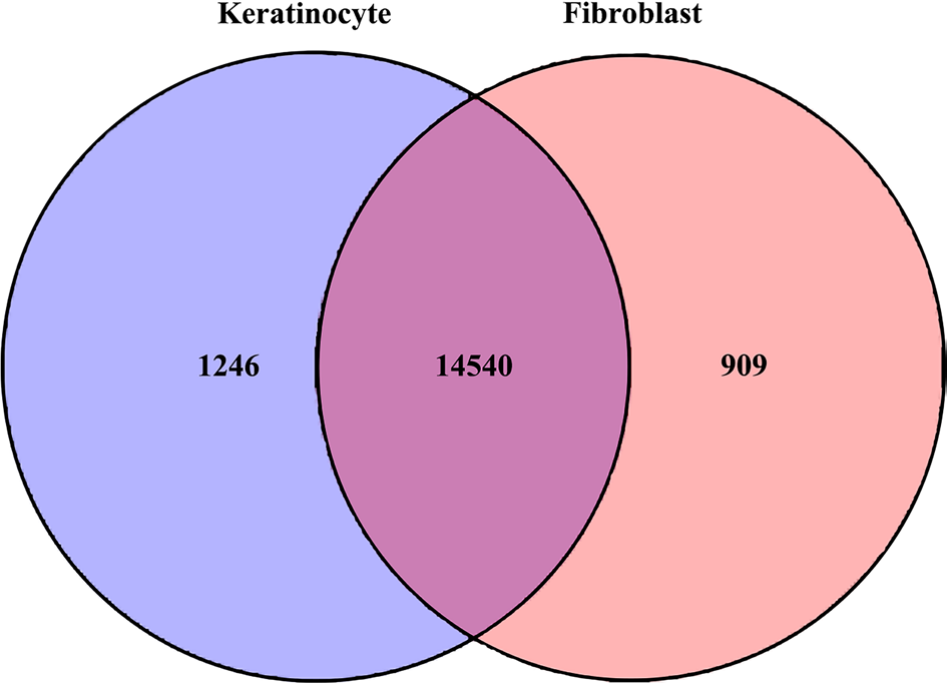
Summary of the numbers of differentially expressed genes. Venn diagram showing the number of genes shared and unique to each cell type

In order to analyze the DEGs between keratinocytes and fibroblasts, we performed the RNA-seq analysis. The results showed that an average of 15,738 genes were detected in each sample, of which a total of 4309 DEGs were identified between keratinocytes and fibroblasts. Among DEGs, 2197 genes were highly expressed in fibroblasts, while 2112 genes were highly expressed in keratinocytes. A Venn diagram of the DEGs indicated that 14,540 genes were shared between the keratinocytes and fibroblasts, 1246 genes were unique to keratinocytes, and 909 genes were unique to fibroblasts (Fig. 2). Based on these data, 36 genes specific to fibroblasts and 205 genes specific to keratinocytes were selected as potential candidate biomarker genes that contribute to distinguishing keratinocytes from fibroblasts (Additional file 3: Table S3 and Additional file 4: Table S4). In this study, FPKM values ≥ 1 indicated the expressed genes, while FPKM values < 0.05 indicated gene expression levels close to zero.

### Enriched GO classification analysis

**Fig. 3:**
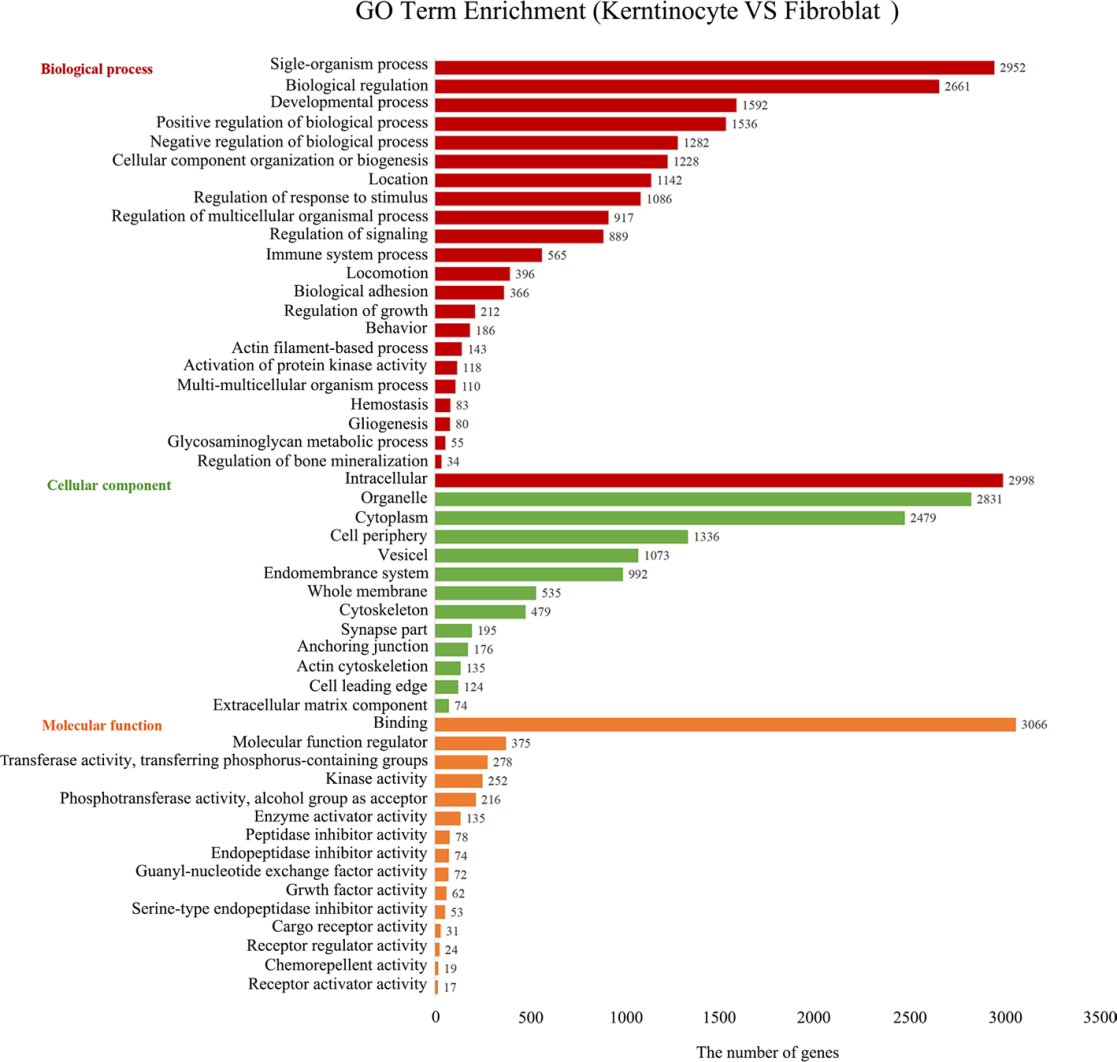
GO enrichment analysis of DEGs. In order to determine the main biological function of the DEGs, GO enrichment analysis was carried out. The X axis represents the number of differentially expressed genes, and the Y axis represents the category (biological process, cellular component, and molecular function). Each column represents a subcategory of the corresponding category

In order to better understand the functions of DEGs, we used Gene Ontology (GO) enrichment analysis, which is the most widely used scheme for classifying gene functions [21]. Through GO enrichment analysis, we can roughly know for which biological functions, pathways or cell locations the DEGs are enriched. GO enrichment analysis classified DEGs into three categories: biological process, cellular component, and molecular function. As shown in Fig. 3, biological process classification showed that the DEGs were mainly mapped into the GO terms single-organism process (2952 genes), biological regulation (2661 genes), development process (1592 genes), positive regulation of biological process (1536 genes), positive regulation of cellular process (1322 genes), negative regulation of biological process (1282 genes), cellular component organization or biogenesis (1228 genes), and localization (1142 genes). Cellular component classification showed that the DEGs were mainly mapped into the GO terms intracellular (2998 genes), organelle (2831 genes), cytoplasm (2479 genes), cell periphery (1336 genes), and vesicle (1073 genes). Molecular function classification showed that the DEGs were mainly mapped into the GO terms binding (3066 genes), molecular function regulator (375 genes), transferase activity, transferring phosphorus-containing group (278 genes), kinase activity (252 genes), phosphotransferase activity, and alcohol group as acceptor (216 genes).

### Identification of differentially expressed collagen genes

Collagen in the skin is an important component for maintaining the skin’s elasticity and mediating wound healing. Collagen aging is one of the factors leading to skin wrinkles. Therefore, understanding the difference in collagen expression between keratinocytes and fibroblasts is very important for antiaging research. Based on the transcriptomics data, a subset of specifically expressed collagen genes in keratinocytes and fibroblasts was identified (Additional file 5: Table S5). Fibroblasts are responsible for the tensile strength of the skin tissue as they synthesize various connective tissue matrix proteins. Thus, the expression levels of 10 collagen genes, such as Col1a1 (collagen, type I, alpha 1), Col28a1 (collagen, type XXVIII, alpha 1), Col5a2 (collagen, type V, alpha 2), and Col3a1 (collagen, type III, alpha 1), etc., were significantly higher in fibroblasts than in keratinocytes. Seven other types of collagen genes, such as Col8a2 (collagen, type VIII, alpha 2), Col17a1 (collagen, type XVII, alpha 1), Col4a4 (collagen, type IV, alpha 4), and Col2a1 (collagen, type II, alpha 1), etc., were more highly expressed in keratinocytes.

### Identification of differentially expressed transcription factors

Among the differentially expressed genes in keratinocytes and fibroblasts, a total of 30 transcription factors were screened. As shown in Additional file 6: Table S6, 14 transcription factors, such as Tfap2e (transcription factor AP-2, epsilon) and Sp6 (trans-acting transcription factor 6), were highly expressed in keratinocytes, while 16 transcription factors, such as Atoh8 (atonal bHLH transcription factor 8), Twist2 (twist basic helix-loop-helix transcription factor 2), and Tcfl5 (transcription factor-like 5 (basic helix-loop-helix)), were highly expressed in fibroblasts.

### Identification of differentially expressed growth factors

Growth factors have a wide range of regulatory functions in the growth and development of the body. As shown in Additional file 7: Table S7, among the DEGs between keratinocytes and fibroblasts, a total of 22 types of growth factors were selected. Fibroblasts showed high expression of 14 types of growth factors, such as Fgf (fibroblast growth factor), Ctgf (connective tissue growth factor), Hgf (hepatocyte growth factor), and Vegfd (vascular endothelial growth factor D). Keratinocytes highly expressed eight types of growth factors, such as Pdgfb (platelet-derived growth factor, B polypeptide), Hbegf (heparin-binding EGF-like growth factor), and Tgfbi (transforming growth factor, beta induced).

### KEGG pathway analysis

**Fig. 4:**
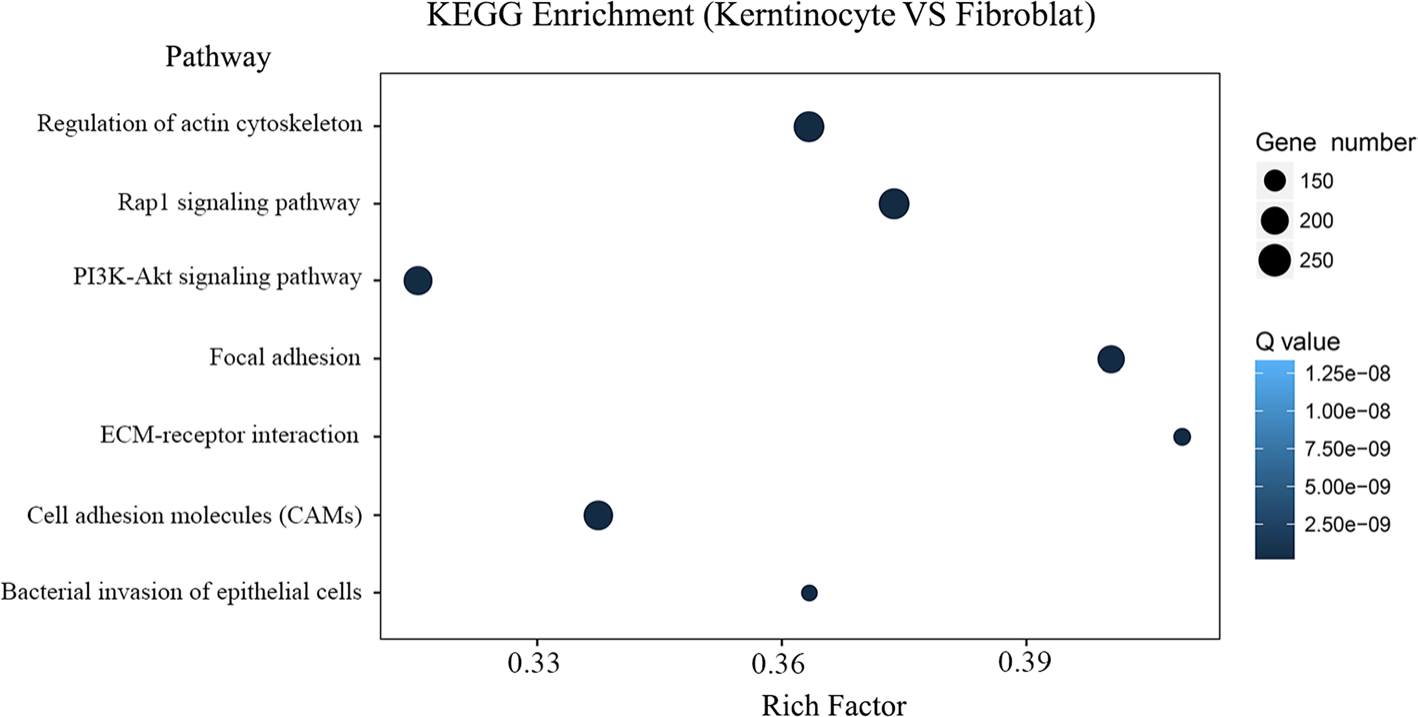
Scatter plot of KEGG enrichment of differential genes. To investigate the main functional pathway of DEGs, KEGG enrichment analysis was carried out. The vertical axis represents the name of the pathway, the horizontal axis represents the rich factor, and the dot size indicates the number of differentially expressed genes in this pathway. The color of the points corresponds to different Q value ranges. Rich factor refers to the ratio of the number of differential genes (sample number) enriched in the pathway to the number of annotation genes (background number). A greater Rich factor value indicates greater enrichment

To further investigate the biological pathways that were involved in the DEGs, we performed KEGG analysis on DEGs. DEGs between keratinocytes and fibroblasts mainly mapped into seven pathways, including regulation of actin cytoskeleton, rap1 signaling pathway, PI3K–Akt signaling pathway, focal adhesion, ECM-receptor interaction, cell adhesion molecules, and bacterial invasion of epithelial cells (Fig. 4).

### Verification of DEGs using qRT-PCR

**Fig. 5:**
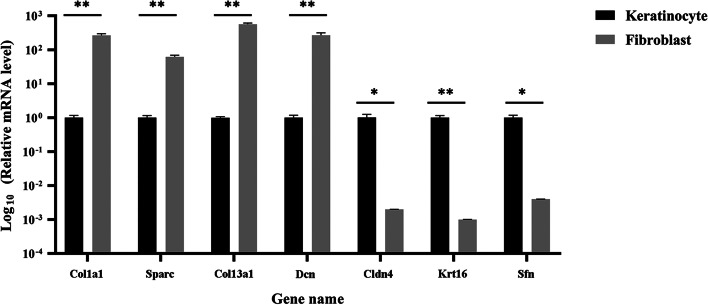
Validation of skin disease related genes in RNA-Seq data using qRT-PCR. Seven skin disease related genes were selected to be analyzed by qRT-PCR. The relative mRNA expression was expressed as 2^−ΔΔCT^. The Gapdh gene served as an internal reference to normalize the target gene expression. Mean ± SD (n = 3). *P < 0.05, **P < 0.01

To verify whether the transcriptome data were reliable, we performed qRT-PCR to confirm the gene expression levels in keratinocytes and fibroblasts. Ten DEGs were randomly selected. Among them, five genes—Plac9a, Ccl12 (chemokine (C–C motif) ligand 12), Car3 (carbonic anhydrase 3), Plac8 (placenta-specific 8), and Agtr2 (angiotensin II receptor, type 2)—were highly expressed in keratinocytes, and five genes—Lce3c (late cornified envelope 3C), Ccer2 (coiled-coil glutamate-rich protein 2), Cfn (cornifelin), Sprr2h, and Prnd (prion protein doublet)—were highly expressed in fibroblasts. The primers were designed based on RNA-Seq data and are listed in Additional file 8: Table S8. The relative fold change was calculated by normalizing to Gapdh, an internal reference gene. As shown in Additional file 9: Figure S1, the qRT-PCR results showed that Plac9a, Ccl12, Car3, Plac8, and Agtr2 were highly expressed in keratinocytes, while Lce3c, Ccer2, Cfn, Sprr2h, and Prnd were highly expressed in fibroblasts, which means that each gene determined by qRT-PCR is similar to the gene observed by RNA-seq. Additionally, seven disease-related genes—skin wound healing-related genes Col1a1, Col13a1, Sparc (secreted protein acidic and rich in cysteine), Dcn, genetic skin disorders-related gene Krt16 (keratin16), skin barrier function-related gene Sfn (stratifin), and Cldn4 (claudin-4)—were detected by qRT-PCR. The primers were designed based on RNA-Seq data and are listed in Additional file 8: Table S8. The results showed that the wound healing-related genes Col1a1, Sparc and Col13a1 were highly expressed in fibroblasts and Krt16, Sfn and Cldn4 were highly expressed in keratinocytes (Fig. 5).

## Discussion

The skin is the largest organ of the human body. In addition to protecting the underlying soft tissues, the skin also has other functions, such as protection against injuries, bacterial attacks, and water loss [22]. The skin consists of many cell types, among which keratinocytes and fibroblasts are considered to be the most abundant. The characteristics of different cells are related to the cell gene expression pattern. Therefore, to understand the characteristics of keratinocytes and fibroblasts, it is necessary to identify genes whose expression is limited to specific cells. To examine gene expression profiles, multiple methods have been developed, including DNA microarray technology and RNA-seq technology. Compared with the microarray technology, RNA-seq has several advantages, including higher sensitivity and a wider range of detection, especially in detecting low-abundance transcripts and discovering new genes [23, 24].

Using RNA-seq technology, we provided a global overview of the gene expression profiles in primary keratinocytes and fibroblasts. We compared the transcriptomes of keratinocytes with fibroblasts to identify the keratinocyte- and fibroblast-specific genes. In our study, an average of 15,738 genes were detected in each sample and a total of 4309 DEGs were screened. There were 2197 genes highly expressed in fibroblasts and 2122 genes highly expressed in keratinocytes. Further analysis of the transcriptome data revealed that 14,540 genes were shared between keratinocytes and fibroblasts, while 1246 and 909 genes were unique to keratinocytes and fibroblasts, respectively. Among them, keratins have been reported to be specifically expressed in keratinocytes, such as keratin 1, keratin 15, keratin 10, and keratin 14. In this study, we used anti-Krt15 antibodies to measure the purity of keratinocytes. Furthermore, it was previously also reported that loricrin was detected in human keratinocytes by Western blot [25]. Consistent with previous reports, we also found that the gene is highly expressed in primary mouse keratinocytes. SFRP2, vimentin and FSP1 have been used as markers for the detection of fibroblasts. For example, SFRP2 was used to confirm the major fibroblast population by immunofluorescence [26]. In this study, FSP1 was used to identify the purity of fibroblasts. In addition, we found that some new markers, such as Plac8, Agtr2, Serping1, Ly6c1 and Dpt, were specifically expressed in fibroblasts, while Sprr1a, Lce3c, Ccer2, Col18a1, Col17a1, Sprr2h and Prnd were specifically expressed in keratinocytes. These genes can be used as novel markers for identification of keratinocytes and fibroblasts.

Collagen is critical for maintaining the elasticity, hydration, and health of the skin. Collagen is mainly distributed in the dermis, such as Col1a1 (collagen type I, alpha 1), Col3a1 (collagen type III, alpha 1), and Col5a2 (collagen type V, alpha 2). Among the collagen types, Col1a1 is the major structural extracellular matrix (ECM) protein, and is most abundant in the skin [27]. As expected, we found that the Col1a1 gene was highly expressed in fibroblasts. The Col3a1 gene was also highly expressed in fibroblasts, and it encodes type III collagen, which is an important protein for maintaining skin balance together with type I collagen [28]. The main characteristics of skin aging are thinning of the dermis layer, the decrease of collagen, and the structural changes in the extracellular matrix leading to wrinkles. Increasing specific collagen may improve skin elasticity and delay skin aging. Therefore, those DEGs that encode collagens may provide therapeutic targets for skin aging.

The epidermis is mainly composed of multiple layers of regenerated keratinocytes. It forms the epidermal barrier and contributes to the defense response to various environmental stressors. By constantly replenishing keratinocytes in the outermost layer of the epidermis, the skin constantly renews itself throughout life. Transcription factors that regulate transcription of a variety of genes and play an important role in cell proliferation and differentiation may participate in regulating skin renewal. Our analysis showed that 14 transcription factors were highly expressed in keratinocytes, while 16 transcription factors were highly expressed in fibroblasts. In this study, Sp6 was highly expressed in keratinocytes and poorly expressed in fibroblasts. It has been reported that low expression of Sp6 can promote proliferation of keratinocyte progenitor cells, while high Sp6 expression promotes cell cycle withdrawal and induces differentiation through Notch 1 [29]. Thus, we speculated that Sp6, as a transcriptional factor, may play a role in the regulation of keratinocyte differentiation and fibroblast proliferation, suggesting that Sp6 may be a potential therapeutic target for wound healing. Keratinocytes highly express ATF3, a member of the transcription factor ATF/cyclic AMP response element binding protein family, which is involved in regulation of the immune response, apoptosis, DNA repair, and tumorigenesis. It has been reported that expression of ATF3 in inflammatory dermatoses can be increased and regulated by IFN-γ in human keratinocytes [30]. Additionally, ATF3 has been reported to promote tumor cell proliferation; however, it is still unknown whether it can regulate proliferation of keratinocytes. In addition, a few studies have examined transcription factors such as Twist2, Atoh8, and Pou3f1 in keratinocytes or fibroblasts. In this study we investigated the expression levels of transcription factors in keratinocytes and fibroblasts, and future studies should try to investigate the functions of these transcription factors.

In the study, we also identified 22 differentially expressed growth factors. Among them, Fgf has a role in the regulation of important signaling pathways and induction of type I collagen synthesis. Moreover, it plays an important role in cell regeneration and repair. Several reports have indicated that Fgf-2 plays an important role in regulating normal processes, such as angiogenesis, wound healing, and tissue repair, and it has antiaging effects [31, 32]. The aging of the dermis is the beginning of the real aging of the skin; Fgf-2 was highly expressed in fibroblasts of newborn mouse skin. Therefore, Fgf-2 has potential as a candidate for anti-skin aging research. Connective tissue growth factor (Ctgf, also known as CCN2) plays an important role in many biological processes, including cell adhesion, migration, proliferation, tissue wound repair, and extracellular matrix remodeling. Blocking CTGF with human anti-CTGF antibody can effectively prevent the development of skin fibrosis, and deletion of CTGF from fibroblasts and smooth muscle cells/pericytes prevents the increase of collagen deposition and muscle fibroblast accumulation in the skin [33]. Additionally, it has been reported that when the epidermis is regenerated, keratinocytes can down-regulate the activity of Ctgf in fibroblasts [34]. In the present study, we found that Ctgf was highly expressed in keratinocytes and poorly expressed in fibroblasts. This suggests that high expression of Ctgf in keratinocytes is also necessary for skin development.

By comparing the gene expression of keratinocytes and fibroblasts, we can not only understand the difference in gene expression in these two cells, but also know where the genes related to disease are expressed, thus providing references for targeted therapies. For example, Lce3c, Cnfn and Krt16 are associated with psoriasis, and Calm4 can be used for the formation, maintenance and repair of the epidermal barrier. In this study, we found that these genes are mainly expressed in keratinocytes. Sfrp2 functions as a melanin irritant and plays a role in the development of ultraviolet-induced allergic diseases. Col1a1 and Col3a1 are related to wound repair, and these genes are expressed more in fibroblasts. It is well known that while genetic identity between humans and mice is more than 95%, the mouse genome is 14% smaller than that of humans, and about 40% of the human genome can be aligned with the mouse genome. Mouse genome sequencing is an important information tool for understanding human genome content, and also a key experimental tool for biomedical research [35]. Furthermore, skin healing is similar in humans and mice [36]. Therefore, although there are differences in immunity and heredity between humans and mice, our findings in this study using a mouse model could provide valuable guidance for understanding the repair of normal and pathological human skin.

## Conclusions

In summary, we conducted comprehensive transcriptome sequencing of keratinocytes and fibroblasts and found a substantial list of novel marker genes for discriminating between keratinocytes and fibroblasts. Our data provided detailed gene expression information about the two cell types, which laid the foundation for further study of the skin cell characteristics. Through further analysis of RNA-seq data, DEGs of collagen, transcription factors, and growth factors were detected, such as Col3a1, Col1a1, Sp6, ATF3, and Fgf-2. These genes play functional roles in maintaining structural integrity and function and regulating cell proliferation. These results provide potential targets for the treatment of skin-related disorders. Additionally, some new genes were identified, but the functions of these genes remain to be further determined.

## Supplementary Information

**Additional file 1: Table S1.** Primers sequences used to detect known genes of keratinocyte and fibroblast by qRT-PCR.

**Additional file 2: Table S2. **Statistics of sequencing and assembly results.

**Additional file 3: Table S3.** Fibroblast-specific genes.

**Additional file 4:Table S4.** Keratinocyte-specific genes.

**Additional file 5: Table S5.** Gene expression levels of collagen genes.

**Additional file 6: Table S6.** Gene expression levels of transcription factor.

**Additional file 7: Table S7. **Gene expression levels of growth factor.

**Additional file 8: Table S8. **Primers sequences used for qRT-PCR to validate the RNA-Seq results.

**Additional file 9: Figure S1.** Validation of RNA-Seq data using qRT-PCR. Ten genes were randomly selected to be analyzed by qRT-PCR. The relative mRNA expression was expressed as 2^−ΔΔCT^. The Gapdh gene served as an internal reference to normalize the target gene expression. Mean ± SD (n = 3). *P < 0.05, **P < 0.01

## Data Availability

The data from this study are available from the author for correspondence on reasonable request.

## Abbreviations

RNA-Seq: RNA sequencing
DEGs: Differentially expressed genes
FDR: False discovery rate
qRT-PCR: Quantitative real time PCR
FPKM: Fragments per kilobase of transcript per million fragments mapped
GO: Gene Ontology
KEGG: Kyoto Encyclopedia of Genes and Genomes
