# Supplementary material for: Comprehensive RNA sequencing in primary murine keratinocytes and fibroblasts identifies novel biomarkers and provides potential therapeutic targets for skin-related diseases
Source: Cell Mol Biol Lett. 2021 Oct 3;26:42. doi: 10.1186/s11658-021-00285-6 (PMC8489068; doi:10.1186/s11658-021-00285-6)
Supplement: Supplementary file 1 — Additional file 1: Table S1. Primers sequences used to detect known genes of keratinocyte and fibroblast by qRT-PCR. [file 11658_2021_285_MOESM1_ESM.docx]

**Table S1.** Primers sequences used to detect known genes of keratinocyte and fibroblast by qRT-PCR

| Gene | Primer | Sequence |
| --- | --- | --- |
| Krt1 | Forward | TGGGAGATTTTCAGGAGGAGG |
|  | Reverse | GCCACACTCTTGGAGATGCTC |
| Krt2 | Forward | GGGCTTCAGTAGCGGTTCAG |
|  | Reverse | ACTAGAGATGCTCTTGTACCCG |
| Krt5 | Forward | TCTGCCATCACCCCATCTGT |
|  | Reverse | CCTCCGCCAGAACTGTAGGA |
| Krt10 | Forward | GCCTCCTACATGGACAAAGTC |
|  | Reverse | GCTTCTCGTACCACTCCTTGA |
| Krt14 | Forward | AGCGGCAAGAGTGAGATTTCT |
|  | Reverse | CCTCCAGGTTATTCTCCAGGG |
| Krt15 | Forward | AGCTATTGCAGAGAAAAACCGT |
|  | Reverse | GGTCCGTCTCAGGTCTGTG |
| Cd90 | Forward | TGCTCTCAGTCTTGCAGGTG |
|  | Reverse | TGGATGGAGTTATCCTTGGTGTT |
| Dcn | Forward | TCTTGGGCTGGACCATTTGAA |
|  | Reverse | CATCGGTAGGGGCACATAGA |
| Dik1 | Forward | CCCAGGTGAGCTTCGAGTG |
|  | Reverse | GGAGAGGGGTACTCTTGTTGAG |
| Lum | Forward | CTCTTGCCTTGGCATTAGTCG |
|  | Reverse | GGGGGCAGTTACATTCTGGTG |
| PDGFRα | Forward | TCCATGCTAGACTCAGAAGTCA |
|  | Reverse | TCCCGGTGGACACAATTTTTC |
| PDGFRβ | Forward | TTCCAGGAGTGATACCAGCTT |
|  | Reverse | AGGGGGCGTGATGACTAGG |
| GAPDH | Forward | GCACAGTCAAGGCCGAGAAT |
|  | Reverse | GCCTTCTCCATGGTGGTGAA |
