# Supplementary material for: Comprehensive RNA sequencing in primary murine keratinocytes and fibroblasts identifies novel biomarkers and provides potential therapeutic targets for skin-related diseases
Source: Cell Mol Biol Lett. 2021 Oct 3;26:42. doi: 10.1186/s11658-021-00285-6 (PMC8489068; doi:10.1186/s11658-021-00285-6)
Supplement: Supplementary file 2 — Additional file 2: Table S2. Statistics of sequencing and assembly results. [file 11658_2021_285_MOESM2_ESM.doc]

| Sample | Clean Reads | Q30 (%) | GC (%) | Total Mapped |
| --- | --- | --- | --- | --- |
| Keratinocyte | 47,468,704 | 93.34 | 52.99 | 44,905,144 (94.60%) |
| Fibroblast | 50,373,090 | 93.47 | 52.32 | 47,817,082 (94.93%) |

**Table S2.** Statistics of sequencing and assembly results
