# Supplementary material for: Comprehensive RNA sequencing in primary murine keratinocytes and fibroblasts identifies novel biomarkers and provides potential therapeutic targets for skin-related diseases
Source: Cell Mol Biol Lett. 2021 Oct 3;26:42. doi: 10.1186/s11658-021-00285-6 (PMC8489068; doi:10.1186/s11658-021-00285-6)
Supplement: Supplementary file 3 — Additional file 3: Table S3. Fibroblast-specific genes. [file 11658_2021_285_MOESM3_ESM.docx]

| Gene name | Gene Expression (FPKM**^#^**) | | log2 Fold Change (Fibroblast/Keratinocyte) | FDR |
| --- | --- | --- | --- | --- |
|  | Keratinocyte | Fibroblast |  |  |

**Table S3.** Fibroblast-specific genes

| Placenta specific 9a (Plac9a) | 0.01 | 28.37 | 11.47 | 5.35E-29 |
| --- | --- | --- | --- | --- |
| Angiotensin II receptor, type 2 (Agtr2) | 0.04 | 17.39 | 8.76 | 2.09E-148 |
| Chemokine (C-C motif) ligand 8 (Ccl8) | 0.01 | 15.19 | 10.57 | 2.47E-17 |
| Chemokine (C-C motif) ligand 12 (Ccl12) | 0.01 | 15.10 | 10.56 | 3.48E-18 |
| Carbonic anhydrase 3 (Car3) | 0.01 | 8.80 | 9.78 | 1.06E-38 |
| Major urinary protein LOC100048885 (Gm2083) | 0.01 | 8.12 | 9.67 | 9.39E-19 |
| Interleukin 31 receptor A (Il31ra) | 0.01 | 5.85 | 9.19 | 2.89E-94 |
| CD300 molecule like family member D5 (Cd300ld5) | 0.01 | 4.64 | 8.86 | 1.05E-09 |
| ATP-binding cassette, sub-family A (ABC1), member 9 (Abca9) | 0.02 | 4.24 | 7.73 | 1.18E-80 |
| Major urinary protein 7 (Mup7) | 0.01 | 4.08 | 8.67 | 7.81E-11 |
| Major urinary protein 14 (Mup14) | 0.01 | 4.06 | 8.67 | 5.48E-10 |
| Major urinary protein 19 (Mup19) | 0.01 | 3.62 | 8.50 | 2.66E-08 |
| Major urinary protein 15 (Mup15) | 0.01 | 3.38 | 8.40 | 5.06E-08 |
| Major urinary protein 3 (Mup3) | 0.01 | 3.28 | 8.36 | 2.66E-08 |
| Major urinary protein 20 (Mup20) | 0.01 | 3.15 | 8.30 | 9.65E-08 |
| Basic helix-loop-helix family, member e22 (Bhlhe22) | 0.03 | 2.79 | 6.54 | 1.26E-25 |
| Protein phosphatase 1, regulatory (inhibitor) subunit 3C (Ppp1r3c) | 0.01 | 2.65 | 8.05 | 1.36E-21 |
| GTPase, very large interferon inducible 1 (Gvin1) | 0.01 | 2.57 | 8.01 | 6.70E-72 |
| Collagen, type XXVIII, alpha 1 (Col28a1) | 0.01 | 2.49 | 7.96 | 2.02E-32 |
| Major urinary protein 11 (Mup11) | 0.01 | 2.46 | 7.94 | 4.61E-06 |
| CD300 molecule like family member D4 (Cd300ld4) | 0.01 | 2.21 | 7.79 | 0 |
| Predicted gene 4951 (Gm4951) | 0.01 | 2.20 | 7.78 | 2.62E-21 |
| Fibroblast growth factor 23 (Fgf23) | 0.04 | 2.19 | 5.77 | 1.99E-16 |
| Follagen, type VI, alpha 6 (Col6a6) | 0.01 | 2.05 | 7.68 | 2.14E-43 |
| Placenta-specific 8 (Plac8) | 2.51 | 255.94 | 6.67 | 0 |
| Serpingne (or cysteine) peptidase inhibitor, clade G, member 1 (Serping1) | 3.24 | 167.52 | 5.69 | 0 |
| Ly6chocyte antigen 6 complex, locus C1 (Ly6c1) | 6.91 | 221.7 | 5.00 | 0 |
| Dermatopontin (Dpt) | 16.23 | 399.2 | 4.62 | 0 |
| Secreted frizzled-related protein 2 (Sfrp2) | 13.42 | 316.29 | 4.56 | 0 |
| Decorin (Dcn) | 12.17 | 299.25 | 4.62 | 0 |
| Twist basic helix-loop-helix transcription factor 2 (Twist2) | 11.79 | 188.94 | 4.00 | 0 |
| Serine (or cysteine) peptidase inhibitor, clade G, member 1 (Serping1) | 3.24 | 167.52 | 5.69 | 0 |
| Delta-like 1 homolog (Drosophila) (Dlk1) | 2.67 | 194.86 | 6.19 | 0 |
| Twist basic helix-loop-helix transcription factor 1 (Twist1) | 19.54 | 146.16 | 2.90 | 0 |
| Lipoprotein lipase (Lpl) | 6.79 | 160.84 | 4.57 | 0 |
| Ly6ahocyte antigen 6 complex, locus A(Ly6a) | 68.94 | 1031.51 | 3.90 | 0 |

**#**Gene expression levels were measured using the FPKM method. FPKM, fragments per kilobase of transcript per million fragments mapped
