# Supplementary material for: Comprehensive RNA sequencing in primary murine keratinocytes and fibroblasts identifies novel biomarkers and provides potential therapeutic targets for skin-related diseases
Source: Cell Mol Biol Lett. 2021 Oct 3;26:42. doi: 10.1186/s11658-021-00285-6 (PMC8489068; doi:10.1186/s11658-021-00285-6)
Supplement: Supplementary file 4 — Additional file 4:Table S4. Keratinocyte-specific genes. [file 11658_2021_285_MOESM4_ESM.docx]

| Gene name | Gene Expression (FPKM**^#^**) | | log2 Fold Change (Fibroblast/Keratinocyte) | FDR |
| --- | --- | --- | --- | --- |
|  | Keratinocyte | Fibroblast |  |  |

**Table S4.** Keratinocyte-specific genes

| Late cornified envelope 3C (Lce3c) | 389.55 | 0.01 | -15.25 | 0 |
| --- | --- | --- | --- | --- |
| Coiled-coil glutamate-rich protein 2 (Ccer2) | 215.99 | 0.01 | -14.40 | 0 |
| Cornifelin (Cnfn) | 197.49 | 0.01 | -14.27 | 2.61E-296 |
| Small proline-rich protein 2H (Sprr2h) | 152.90 | 0.01 | -13.90 | 5.37E-296 |
| Defensin beta 14 (Defb14) | 84.60 | 0.01 | -13.05 | 2.40E-46 |
| Prion protein dublet (Prnd) | 83.48 | 0.01 | -13.03 | 0 |
| Small proline-rich protein 2G (Sprr2g) | 82.34 | 0.01 | -13.01 | 7.73E-132 |
| Late cornified envelope 1F (Lce1f) | 77.73 | 0.01 | -12.92 | 2.04E-154 |
| Lymphocyte antigen 6 complex, locus G6C (Ly6g6c) | 76.08 | 0.01 | -12.89 | 2.69E-199 |
| Cysteine rich tail 1 (Cysrt1) | 71.16 | 0.01 | -12.80 | 2.23E-144 |
| Small proline-rich protein 2D (Sprr2d) | 68.53 | 0.01 | -12.74 | 1.77E-116 |
| Late cornified envelope 3E (Lce3e) | 68.06 | 0.01 | -12.73 | 6.37E-99 |
| Late cornified envelope 1A1 (Lce1a1) | 67.73 | 0.01 | -12.73 | 8.40E-122 |
| Late cornified envelope 3F (Lce3f) | 60.46 | 0.01 | -12.56 | 1.02E-91 |
| Calpain, small subunit 2 (Capns2) | 54.72 | 0.01 | -12.42 | 3.68E-153 |
| Small proline-rich protein 2F (Sprr2f) | 45.59 | 0.01 | -12.15 | 1.14E-66 |
| Trefoil factor 3, intestinal (Tff3) | 43.40 | 0.01 | -12.08 | 5.68E-42 |
| Kallikrein related-peptidase 11 (Klk11) | 42.32 | 0.01 | -12.05 | 1.94E-159 |
| Cell death-inducing DNA fragmentation factor, alpha subunit-like effector A (Cidea) | 42.08 | 0.01 | -12.04 | 2.02E-138 |
| Small proline-rich protein 2A3 (Sprr2a3) | 39.07 | 0.01 | -11.93 | 2.85E-88 |
| Chloride intracellular channel 3 (Clic3) | 36.89 | 0.01 | -11.85 | 5.39E-77 |
| Kallikrein related-peptidase 5 (Klk5) | 34.95 | 0.01 | -11.77 | 4.75E-139 |
| S100 calcium binding protein A9 (calgranulin B) (S100a9) | 27.39 | 0.01 | -11.42 | 1.50E-33 |
| S100 calcium binding protein A8 (calgranulin A) (S100a8) | 25.04 | 0.01 | -11.29 | 6.23E-19 |
| Ribosomal protein S27, retrogene (Rps27rt) | 24.41 | 0.01 | -11.25 | 9.20E-17 |
| Late cornified envelope 1A2 (Lce1a2) | 24.37 | 0.01 | -11.25 | 3.68E-44 |
| Late cornified envelope 1D (Lce1d) | 24.27 | 0.01 | -11.24 | 4.90E-41 |
| Three prime repair exonuclease 2 (Trex2) | 21.45 | 0.01 | -11.07 | 2.39E-61 |
| Late cornified envelope 1H (Lce1h) | 20.74 | 0.01 | -11.02 | 5.55E-37 |
| Late cornified envelope 1E (Lce1e) | 19.22 | 0.01 | -10.91 | 9.80E-36 |
| Keratin 23 (Krt23) | 16.16 | 0.01 | -10.66 | 3.56E-74 |
| Late cornified envelope 3B (Lce3b) | 15.11 | 0.01 | -10.56 | 7.34E-20 |
| Growth factor receptor bound protein 7 (Grb7) | 15.07 | 0.01 | -10.56 | 1.86E-111 |
| Transmembrane protein 125 (Tmem125) | 14.33 | 0.01 | -10.48 | 7.31E-74 |
| RIKEN cDNA 2310050C09 gene (2310050C09Rik) | 13.98 | 0.01 | -10.45 | 6.57E-43 |
| RIKEN cDNA 2610528J11 gene (2610528J11Rik) | 12.68 | 0.01 | -10.31 | 2.26E-31 |
| Ly6/Plaur domain containing 5 (Lypd5) | 12.60 | 0.01 | -10.30 | 5.41E-32 |
| Small proline-rich protein 2I (Sprr2i) | 11.98 | 0.01 | -10.23 | 5.32E-18 |
| Glutathione S-transferase pi 3 (Gstp3) | 11.82 | 0.01 | -10.21 | 1.19E-22 |
| Short chain dehydrogenase/reductase family 16C, member 5 (Sdr16c5) | 11.68 | 0.01 | -10.19 | 1.17E-46 |
| Calmodulin-like 3 (Calml3) | 11.62 | 0.01 | -10.18 | 3.20E-48 |
| Short chain dehydrogenase/reductase family 16C, member 6 (Sdr16c6) | 11.61 | 0.01 | -10.18 | 7.47E-39 |
| Casitas B-lineage lymphoma c (Cblc) | 11.38 | 0.01 | -10.15 | 4.92E-56 |
| Interleukin 17 receptor E (Il17re) | 11.16 | 0.01 | -10.12 | 6.75E-89 |
| Serine (or cysteine) peptidase inhibitor, clade A (alpha-1 antiproteinase, antitrypsin), member 9 (Serpina9) | 11.12 | 0.01 | -10.12 | 1.35E-57 |
| Late cornified envelope 1I (Lce1i) | 11.09 | 0.01 | -10.12 | 8.62E-21 |
| Potassium channel, subfamily K, member 7 (Kcnk7) | 11.05 | 0.01 | -10.11 | 9.81E-36 |
| Late cornified envelope 1B (Lce1b) | 10.85 | 0.01 | -10.08 | 2.22E-17 |
| Prostate stem cell antigen (Psca) | 10.83 | 0.01 | -10.08 | 1.89E-25 |
| Small integral membrane protein 22 (Smim22) | 10.55 | 0.01 | -10.04 | 5.66E-10 |
| Bone gamma-carboxyglutamate protein 3 (Bglap3) | 10.40 | 0.01 | -10.02 | 1.49E-19 |
| Hemoglobin, beta adult s chain (Hbb-bs) | 10.28 | 0.01 | -10.01 | 3.82E-16 |
| Zinc finger protein 750 (Zfp750) | 30.84 | 0.03 | -10.01 | 3.44E-311 |
| Forkhead box N1 (Foxn1) | 9.99 | 0.01 | -9.96 | 1.98E-101 |
| Family with sequence similarity 84, member A (Fam84a) | 9.29 | 0.01 | -9.86 | 3.10E-99 |
| Keratin 75 (Krt75) | 26.04 | 0.03 | -9.76 | 1.58E-263 |
| MHC I like leukocyte 1 (Mill1) | 8.60 | 0.01 | -9.75 | 9.91E-66 |
| Transformation related protein 63 regulated (Tprg) | 8.59 | 0.01 | -9.75 | 4.49E-26 |
| Transient receptor potential cation channel, subfamily M, member 1 (Trpm1) | 8.47 | 0.01 | -9.73 | 5.39E-77 |
| Connector enhancer of kinase suppressor of Ras 1 (Cnksr1) | 33.44 | 0.04 | -9.71 | 5.44E-251 |
| Keratin 78 (Krt78) | 8.30 | 0.01 | -9.70 | 3.28E-89 |
| Epsin 3 (Epn3) | 16.50 | 0.02 | -9.69 | 1.33E-199 |
| Lysophosphatidic acid receptor 5 (Lpar5) | 8.09 | 0.01 | -9.66 | 8.49E-70 |
| Histone cluster 1, H4a (Hist1h4a) | 7.78 | 0.01 | -9.60 | 1.60E-07 |
| Keratin 15 (Krt15) | 7.77 | 0.01 | -9.60 | 8.67E-40 |
| Late cornified envelope 3A (Lce3a) | 7.68 | 0.01 | -9.58 | 8.08E-12 |
| Wingless-type MMTV integration site family, member 3 (Wnt3) | 7.58 | 0.01 | -9.57 | 1.50E-73 |
| Parathyroid hormone-like peptide (Pthlh) | 7.45 | 0.01 | -9.54 | 1.11E-31 |
| Gap junction protein, beta 6 (Gjb6) | 7.41 | 0.01 | -9.53 | 6.55E-48 |
| Uroplakin 3B-like (Upk3bl) | 7.30 | 0.01 | -9.51 | 1.50E-19 |
| Defensin beta 6 (Defb6) | 7.16 | 0.01 | -9.48 | 0 |
| Premature ovarian failure 1B (Pof1b) | 14.21 | 0.02 | -9.47 | 1.13E-172 |
| Ribonuclease, RNase A family, 1 (pancreatic) (Rnase1) | 6.79 | 0.01 | -9.41 | 2.60E-18 |
| Histone cluster 3, H2ba (Hist3h2ba) | 6.71 | 0.01 | -9.39 | 6.61E-07 |
| RIKEN cDNA 8430408G22 gene (8430408G22Rik) | 6.69 | 0.01 | -9.39 | 1.89E-25 |
| Late cornified envelope 1C (Lce1c) | 6.63 | 0.01 | -9.37 | 1.95E-12 |
| Small integral membrane protein 5 (Smim5) | 6.61 | 0.01 | -9.37 | 2.22E-17 |
| Small proline-rich protein 2E (Sprr2e) | 6.51 | 0.01 | -9.35 | 1.37E-10 |
| Desmocollin 2 (Dsc2) | 6.47 | 0.01 | -9.34 | 7.54E-95 |
| Dmmunoglobulin-like domain containing receptor 1 (Ildr1) | 6.44 | 0.01 | -9.33 | 1.01E-60 |
| Sclerostin domain containing 1 (Sostdc1) | 6.42 | 0.01 | -9.33 | 1.50E-33 |
| Absent in melanoma 1-like (Aim1l) | 12.80 | 0.02 | -9.32 | 9.03E-192 |
| MARVEL (membrane-associating) domain containing 3 (Marveld3) | 6.36 | 0.01 | -9.31 | 4.80E-36 |
| Solute carrier family 6 (neurotransmitter transporter), member 14 (Slc6a14) | 6.23 | 0.01 | -9.28 | 5.53E-67 |
| BICD family like cargo adaptor 2(Bicdl2) | 6.12 | 0.01 | -9.26 | 9.81E-36 |
| E74-like factor 3(Elf3) | 5.60 | 0.01 | -9.13 | 3.06E-33 |
| Peptidoglycan recognition protein 4(Pglyrp4) | 5.53 | 0.01 | -9.11 | 1.35E-42 |
| Sodium channel, nonvoltage-gated 1 gamma (Scnn1g) | 5.17 | 0.01 | -9.01 | 1.56E-48 |
| Arachidonate 12-lipoxygenase, 12R type (Alox12b) | 5.10 | 0.01 | -8.99 | 1.14E-36 |
| Late cornified envelope 1M (Lce1m) | 5.08 | 0.01 | -8.99 | 3.97E-12 |
| Lymphocyte antigen 6 complex, locus G (Ly6g) | 4.90 | 0.01 | -8.94 | 1.95E-12 |
| Rhomboid, veinlet-like 2 (Drosophila) (Rhbdl2) | 4.88 | 0.01 | -8.93 | 4.52E-17 |
| Uncharacterized LOC105245869 (LOC105245869) | 4.85 | 0.01 | -8.92 | 1.09E-17 |
| Late cornified envelope 1K (Lce1k) | 4.75 | 0.01 | -8.89 | 3.92E-08 |
| Androgen dependent TFPI regulating protein (Adtrp) | 18.92 | 0.04 | -8.89 | 3.39E-144 |
| Gamma-glutamyltransferase 6 (Ggt6) | 4.57 | 0.01 | -8.84 | 5.82E-23 |
| RIKEN cDNA 2310003N18 gene(2310003N18Rik) | 4.53 | 0.01 | -8.82 | 8.82E-05 |
| Serine peptidase inhibitor, Kazal type 8 (Spink8) | 4.51 | 0.01 | -8.82 | 3.92E-08 |
| Serine (or cysteine) peptidase inhibitor, clade B, member 6c (Serpinb6c) | 4.30 | 0.01 | -8.75 | 2.22E-17 |
| Long non-coding RNA, embryonic stem cells expressed 1 (Lncenc1) | 4.29 | 0.01 | -8.74 | 1.01E-45 |
| Abhydrolase domain containing 11, opposite strand (Abhd11os) | 4.29 | 0.01 | -8.74 | 2.18E-05 |
| Acid phosphatase 7, tartrate resistant (Acp7) | 4.28 | 0.01 | -8.74 | 1.62E-24 |
| WAP four-disulfide core domain 3 (Wfdc3) | 4.21 | 0.01 | -8.72 | 2.33E-09 |
| Ring finger protein 43 (Rnf43) | 4.15 | 0.01 | -8.70 | 8.48E-70 |
| Late cornified envelope 6A (Lce6a) | 4.10 | 0.01 | -8.68 | 9.57E-09 |
| Wingless-type MMTV integration site family, member 10B (Wnt10b) | 16.19 | 0.04 | -8.66 | 2.22E-126 |
| Non-specific cytotoxic cell receptor protein 1 homolog (zebrafish) (Nccrp1) | 4.04 | 0.01 | -8.66 | 2.60E-18 |
| Lymphocyte antigen 6 complex, locus G6E(Ly6g6e) | 4.01 | 0.01 | -8.65 | 4.72E-13 |
| Ovo like zinc finger 2 (Ovol2) | 4.00 | 0.01 | -8.64 | 2.22E-17 |
| Keratin 77 (Krt77) | 3.98 | 0.01 | -8.64 | 1.01E-21 |
| Transcription factor AP-2, epsilon (Tfap2e) | 3.96 | 0.01 | -8.63 | 1.07E-26 |
| Family with sequence similarity 3, member B (Fam3b) | 3.91 | 0.01 | -8.61 | 2.33E-09 |
| SH3 domain containing ring finger 2 (Sh3rf2) | 3.90 | 0.01 | -8.61 | 3.70E-59 |
| Sosondowah ankyrin repeat domain family member B (Sowahb) | 3.80 | 0.01 | -8.57 | 1.17E-46 |
| Rho GTPase activating protein 8 (Arhgap8) | 3.77 | 0.01 | -8.56 | 4.52E-17 |
| Small integral membrane protein 24 (Smim24) | 3.71 | 0.01 | -8.54 | 2.18E-05 |
| Peptidoglycan recognition protein 1 (Pglyrp1) | 3.68 | 0.01 | -8.52 | 1.33E-06 |
| Cystatin A1 (Csta1) | 3.67 | 0.01 | -8.52 | 4.65E-31 |
| Hemoglobin alpha, adult chain 2 (Hba-a2) | 3.52 | 0.01 | -8.46 | 4.39E-05 |
| Cytochrome P450, family 3, subfamily a, polypeptide 13 (Cyp3a13) | 3.41 | 0.01 | -8.41 | 2.27E-31 |
| Melanocortin 1 receptor (Mc1r) | 3.39 | 0.01 | -8.41 | 3.66E-39 |
| Late cornified envelope 1L(Lce1l) | 3.39 | 0.01 | -8.41 | 5.40E-06 |
| Desmoglein 1 alpha (Dsg1a) | 3.36 | 0.01 | -8.39 | 4.27E-60 |
| Myosin, heavy polypeptide 14 (Myh14) | 9.97 | 0.03 | -8.38 | 2.54E-203 |
| REC114 meiotic recombination protein (Rec114) | 3.31 | 0.01 | -8.37 | 3.92E-08 |
| Transmembrane protease, serine 4 (Tmprss4) | 12.99 | 0.04 | -8.34 | 1.13E-87 |
| Rho GTPase activating protein 40 (Arhgap40) | 3.22 | 0.01 | -8.33 | 7.35E-20 |
| Small proline-rich protein 2B (Sprr2b) | 3.20 | 0.01 | -8.32 | 5.40E-06 |
| SRY (sex determining region Y)-box 21 (Sox21) | 6.23 | 0.02 | -8.28 | 2.09E-72 |
| Spectrin beta, non-erythrocytic 2 (Sptbn2) | 8.53 | 0.03 | -8.15 | 7.51E-227 |
| Thrombospondin type laminin G domain and EAR repeats (Tspear) | 2.84 | 0.01 | -8.15 | 2.06E-21 |
| Keratin 20(Krt20) | 2.83 | 0.01 | -8.14 | 9.20E-17 |
| Small proline-rich protein 2J, pseudogene (Sprr2j-ps) | 2.81 | 0.01 | -8.13 | 4.39E-05 |
| Carboxylesterase 2E (Ces2e) | 2.81 | 0.01 | -8.13 | 1.09E-17 |
| Cytochrome P450, family 4, subfamily f, polypeptide 39 (Cyp4f39) | 2.79 | 0.01 | -8.12 | 1.19E-22 |
| Transmembrane protein 265 (Tmem265) | 2.77 | 0.01 | -8.11 | 5.66E-10 |
| Insulinoma-associated 1 (Insm1) | 2.72 | 0.01 | -8.09 | 9.21E-26 |
| Serine palmitoyltransferase, long chain base subunit 3 (Sptlc3) | 2.68 | 0.01 | -8.07 | 5.82E-23 |
| Solute carrier family 45, member 2(Slc45a2) | 7.87 | 0.03 | -8.04 | 7.50E-71 |
| Glycine/arginine rich protein 1 (Grrp1) | 2.62 | 0.01 | -8.03 | 1.15E-09 |
| Desmoglein 1 beta (Dsg1b) | 5.22 | 0.02 | -8.03 | 4.98E-97 |
| Glutathione peroxidase 2 (Gpx2) | 2.52 | 0.01 | -7.98 | 7.93E-08 |
| Phospholipase A2 inhibitor and LY6/PLAUR domain containing (Pinlyp) | 2.52 | 0.01 | -7.98 | 4.39E-05 |
| BPI fold containing family C (Bpifc) | 9.99 | 0.04 | -7.96 | 5.48E-69 |
| Deoxyribonuclease 1-like 3 (Dnase1l3) | 7.43 | 0.03 | -7.95 | 6.43E-89 |
| Rhesus blood group-associated B glycoprotein (Rhbg) | 2.45 | 0.01 | -7.94 | 2.73E-14 |
| Interleukin 17B (Il17b) | 2.44 | 0.01 | -7.93 | 4.39E-05 |
| Involucrin (Ivl) | 2.38 | 0.01 | -7.89 | 5.59E-14 |
| cDNA sequence, BC016579 (BC016579) | 2.35 | 0.01 | -7.88 | 7.81E-16 |
| Interleukin 18 receptor 1 (Il18r1) | 4.70 | 0.02 | -7.88 | 6.78E-35 |
| Sterile alpha motif domain containing 12 (Samd12) | 2.35 | 0.01 | -7.88 | 2.68E-06 |
| Protease, serine 27 (Prss27) | 2.32 | 0.01 | -7.86 | 7.93E-08 |
| Transformation related protein 73 (Trp73) | 2.32 | 0.01 | -7.86 | 4.12E-35 |
| RIKEN cDNA 2010109I03 gene (2010109I03Rik) | 2.27 | 0.01 | -7.83 | 4.72E-13 |
| Keratin associated protein 3-2 (Krtap3-2) | 2.09 | 0.01 | -7.71 | 2.68E-06 |
| Potassium channel tetramerisation domain containing 4 (Kctd4) | 2.07 | 0.01 | -7.69 | 1.50E-19 |
| Serine (or cysteine) peptidase inhibitor, clade A (alpha-1 antiproteinase, antitrypsin), member 11 (Serpina11) | 2.06 | 0.01 | -7.69 | 4.72E-09 |
| Trans-acting transcription factor 6 (Sp6) | 6.18 | 0.03 | -7.69 | 4.02E-67 |
| RIKEN cDNA 4930413F20 gene (4930413F20Rik) | 2.04 | 0.01 | -7.67 | 1.33E-06 |
| Adhesion G protein-coupled receptor F2 (Adgrf2) | 2.01 | 0.01 | -7.65 | 8.63E-21 |
| ATP-binding cassette, sub-family A (ABC1), member 12 (Abca12) | 4.79 | 0.03 | -7.32 | 1.66E-122 |
| Hook microtubule tethering protein 1 (Hook1) | 3.17 | 0.02 | -7.31 | 4.59E-41 |
| Family with sequence similarity 83, member B (Fam83b) | 4.74 | 0.03 | -7.30 | 1.81E-44 |
| Solute carrier family 39 (zinc transporter), member 2 (Slc39a2) | 6.23 | 0.04 | -7.28 | 1.31E-42 |
| Adhesion G protein-coupled receptor F4 (Adgrf4) | 4.63 | 0.03 | -7.27 | 5.43E-42 |
| Retinol dehydrogenase 1 (all trans) (Rdh1) | 2.77 | 0.02 | -7.11 | 7.82E-24 |
| Ets homologous factor (Ehf) | 5.25 | 0.04 | -7.04 | 4.47E-69 |
| Sciellin (Scel) | 3.92 | 0.03 | -7.03 | 1.64E-35 |
| Choline dehydrogenase (Chdh) | 3.91 | 0.03 | -7.03 | 1.12E-65 |
| C-C motif chemokine 27-like (LOC100861978) | 4.93 | 0.04 | -6.95 | 0 |
| Predicted gene 13306 (Gm13306) | 4.93 | 0.04 | -6.95 | 0 |
| Chemokine (C-C motif) ligand 27b (Ccl27b) | 4.93 | 0.04 | -6.95 | 0 |
| Predicted gene 2506 (Gm2506) | 4.93 | 0.04 | -6.95 | 0 |
| POU domain, class 2, transcription factor 3 (Pou2f3) | 4.84 | 0.04 | -6.92 | 8.09E-36 |
| Desmocollin 1 (Dsc1) | 2.33 | 0.02 | -6.86 | 3.34E-35 |
| Ankyrin repeat and kinase domain containing 1 (Ankk1) | 4.64 | 0.04 | -6.86 | 4.72E-37 |
| Anoctamin 9 (Ano9) | 4.31 | 0.04 | -6.75 | 1.35E-38 |
| Interleukin 22 receptor, alpha 1 (Il22ra1) | 2.12 | 0.02 | -6.73 | 1.14E-37 |
| Teneurin transmembrane protein 2 (Tenm2) | 3.50 | 0.04 | -6.45 | 7.25E-103 |
| Patatin-like phospholipase domain containing 1 (Pnpla1) | 3.48 | 0.04 | -6.44 | 5.91E-46 |
| Tyrosinase (Tyr) | 2.44 | 0.03 | -6.35 | 4.37E-45 |
| Transient receptor potential cation channel, subfamily V, member 6 (Trpv6) | 2.33 | 0.03 | -6.28 | 7.19E-20 |
| Alkaline ceramidase 1 (Acer1) | 2.65 | 0.04 | -6.05 | 2.37E-18 |
| Interleukin 18 receptor accessory protein (Il18rap) | 2.40 | 0.04 | -5.91 | 5.05E-34 |
| Small proline-rich protein 1A (Sprr1a) | 2620.33 | 4.59 | -9.16 | 0 |
| Stratifin (Sfn) | 2566.94 | 18.68 | -7.10 | 0 |
| Keratinocyte differentiation associated protein (Krtdap) | 1886.12 | 3.26 | -9.18 | 0 |
| Keratin 16 (Krt16) | 1818.14 | 3.28 | -9.11 | 0 |
| Cysteine-rich C-terminal 1 (Crct1) | 1770.99 | 1.62 | -10.09 | 0 |
| Lymphocyte antigen 6 complex, locus D (Ly6d) | 1264.56 | 2.03 | -9.28 | 0 |
| Dermokine (Dmkn) | 1256.8 | 2.95 | -8.73 | 0 |
| Small proline-rich protein 2A2 (Sprr2a2) | 1109.96 | 6.75 | -7.36 | 0 |
| S100 calcium binding protein A14 (S100a14) | 1049.46 | 6.6 | -7.31 | 0 |
| Suprabasin (Sbsn) | 823.45 | 4 | -7.69 | 0 |
| Calmodulin 4 (Calm4) | 795.35 | 0.58 | -10.42 | 0 |
| Kallikrein related-peptidase 7 (chymotryptic, stratum corneum) (Klk7) | 557.66 | 1.42 | -8.62 | 0 |
| Laminin, gamma 2 (Lamc2) | 530.48 | 8.26 | -6.01 | 0 |
| Tumor-associated calcium signal transducer 2 (Tacstd2) | 490.88 | 1.4 | -8.45 | 0 |
| Claudin 4 (Cldn4) | 425.28 | 2.15 | -7.63 | 0 |
| Annexin A8 (Anxa8) | 418.01 | 3.78 | -6.79 | 0 |
| FXYD domain-containing ion transport regulator 3 (Fxyd3) | 416.68 | 0.99 | -8.72 | 0 |
| Tripartite motif-containing 29 (Trim29) | 408.69 | 1.04 | -8.62 | 0 |
| Late cornified envelope 3D (Lce3d) | 401.8 | 0.23 | -10.77 | 0 |
| Plakophilin 1 (Pkp1) | 399.45 | 2.28 | -7.45 | 0 |
| Premelanosome protein (Pmel) | 398.22 | 1.64 | -7.92 | 0 |

#Gene expression levels were measured using the FPKM method. FPKM, fragments per kilobase of transcript per million fragments mapped.
