# Supplementary material for: Comprehensive RNA sequencing in primary murine keratinocytes and fibroblasts identifies novel biomarkers and provides potential therapeutic targets for skin-related diseases
Source: Cell Mol Biol Lett. 2021 Oct 3;26:42. doi: 10.1186/s11658-021-00285-6 (PMC8489068; doi:10.1186/s11658-021-00285-6)
Supplement: Supplementary file 5 — Additional file 5: Table S5. Gene expression levels of collagen genes. [file 11658_2021_285_MOESM5_ESM.docx]

**Table S5.** Gene expression levels of collagen genes

| Gene name | Gene Expression （FPKM**^#^**） | | log_2_ Fold Change (Fibroblast/Keratinocyte) | FDR |
| --- | --- | --- | --- | --- |
|  | Keratinocyte | Fibroblast |  |  |
| Collagen, type I, alpha 1 (Col1a1) | 569.12 | 4727.69 | 3.05 | 0 |
| Collagen, type III, alpha 1 (Col3a1) | 221.45 | 2462.75 | 3.48 | 0 |
| Collagen, type V, alpha 2 (Col5a2) | 61.09 | 632.76 | 3.37 | 0 |
| Collagen, type XII, alpha 1 (Col12a1) | 40.85 | 315.99 | 2.95 | 0 |
| Collagen, type XVI, alpha 1 (Col16a1) | 34.04 | 77.47 | 1.19 | 9.15E-213 |
| Collagen, type XVIII, alpha 1 (Col18a1) | 218.07 | 28.95 | -2.91 | 0 |
| Collagen, type XIV, alpha 1 (Col14a1) | 1.87 | 19.75 | 3.4 | 8.61E-262 |
| Collagen, type XV, alpha 1 (Col15a1) | 5.26 | 18.04 | 1.78 | 2.15E-90 |
| Collagen, type XXIII, alpha 1 (Col23a1) | 2.65 | 9.81 | 1.89 | 2.70E-57 |
| Collagen, type VII, alpha 1 (Col7a1) | 68.63 | 7.83 | -3.13 | 0 |
| Collagen, type XXVIII, alpha 1 (Col28a1) | 0.01 | 2.49 | 7.96 | 2.02E-32 |
| Collagen, type VI, alpha 1 (Col6a1) | 215.53 | 836.55 | 1.96 | 0 |
| Collagen, type XVII, alpha 1 (Col17a1) | 272.63 | 2 | -7.09 | 0 |
| Collagen, type XXIV, alpha 1 (Col24a1) | 0.4 | 1.24 | 1.63 | 6.82E-09 |
| Collagen, type VIII, alpha 2 (Col8a2) | 6.33 | 0.45 | -3.81 | 7.14E-61 |
| Collagen, type XXVI, alpha 1 (Col26a1) | 7.24 | 0.32 | -4.5 | 1.37E-45 |
| Collagen, type II, alpha 1 (Col2a1) | 0.84 | 0.02 | -5.39 | 5.06E-12 |
| Collagen, type IV, alpha 4 (Col4a4) | 0.54 | 0.01 | -5.75 | 2.53E-12 |

#Gene expression levels were measured using the FPKM method. FPKM, fragments per kilobase of transcript per million fragments mapped
