# Supplementary material for: Comprehensive RNA sequencing in primary murine keratinocytes and fibroblasts identifies novel biomarkers and provides potential therapeutic targets for skin-related diseases
Source: Cell Mol Biol Lett. 2021 Oct 3;26:42. doi: 10.1186/s11658-021-00285-6 (PMC8489068; doi:10.1186/s11658-021-00285-6)
Supplement: Supplementary file 6 — Additional file 6: Table S6. Gene expression levels of transcription factor. [file 11658_2021_285_MOESM6_ESM.docx]

**Table S6.** Gene expression levels of transcription factor

| Gene name | Gene Expression (FPKM**^#^**) | | | log_2_ Fold Change (Fibroblast/Keratinocyte) | FDR |
| --- | --- | --- | --- | --- | --- |
|  | Keratinocyte | | Fibroblast |  |  |
| Twist basic helix-loop-helix transcription factor 2 (Twist2) | 11.79 | 188.94 | | 4 | 0 |
| Twist basic helix-loop-helix transcription factor 1 (Twist1) | 19.54 | 146.16 | | 2.93 | 0 |
| Transcription factor 12 (Tcf12) | 13.98 | 38.17 | | 1.44 | 8.66E-120 |
| Transcription factor 4 (Tcf4) | 2.15 | 16.11 | | 2.9 | 4.40E-188 |
| Atonal bHLH transcription factor 8 (Atoh8) | 0.65 | 14.42 | | 4.47 | 1.14E-79 |
| BCL2-associated transcription factor 1 (Bclaf1) | 6.44 | 13.29 | | 1.04 | 1.31E-30 |
| Paired-like homeodomain transcription factor 2 (Pitx2) | 1.36 | 12.38 | | 3.18 | 8.54E-41 |
| AT hook containing transcription factor 1 (Ahctf1) | 4.32 | 10.03 | | 1.21 | 8.44E-48 |
| Activating transcription factor 3 (Atf3) | 51.14 | 9.61 | | -2.41 | 7.50E-135 |
| Transcription factor-like 5 (basic helix-loop-helix) (Tcfl5) | 1.62 | 7.96 | | 2.29 | 6.27E-23 |
| AT-hook transcription factor (Akna) | 2.42 | 6.82 | | 1.49 | 2.17E-28 |
| Activating transcription factor 2 (Atf2) | 2.69 | 6.35 | | 1.23 | 1.29E-14 |
| General transcription factor II A, 1 (Gtf2a1) | 2.06 | 5.14 | | 1.31 | 1.14E-18 |
| Doublesex and mab-3 related transcription factor 2 (Dmrt2) | 0.48 | 4.08 | | 3.08 | 2.69E-17 |
| Transcription factor EB (Tfeb) | 8.64 | 3.96 | | -1.12 | 2.68E-10 |
| Runt-related transcription factor 1; translocated to, 1 (cyclin D-related) (Runx1t1) | 0.25 | 3.12 | | 3.64 | 1.33E-48 |
| Leucine zipper transcription factor-like 1 (Lztfl1) | 1.54 | 3.09 | | 1 | 4.01E-05 |
| Transcription factor EC (Tfec) | 0.18 | 1.16 | | 2.68 | 0 |
| E2F transcription factor 2 (E2f2) | 2.41 | 0.86 | | -1.48 | 1.68E-07 |
| LIM homeobox transcription factor 1 beta (Lmx1b) | 2.53 | 0.51 | | -2.31 | 0 |
| Transcription factor AP-2 beta (Tfap2b) | 3.47 | 0.26 | | -3.73 | 8.40E-33 |
| POU domain, class 3, transcription factor 1 (Pou3f1) | 20.08 | 0.25 | | -6.32 | 5.54E-173 |
| Transcription factor CP2-like 1 (Tfcp2l1) | 0.5 | 0.18 | | -1.47 | 0 |
| Transcription factor AP-2, alpha (Tfap2a) | 29.07 | 0.17 | | -7.41 | 5.50E-284 |
| Transcription factor AP-2, gamma (Tfap2c) | 16.62 | 0.1 | | -7.37 | 1.36E-139 |
| Paired-like homeodomain transcription factor 1 (Pitx1) | 4.92 | 0.08 | | -5.94 | 1.02E-33 |
| POU domain, class 2, transcription factor 3 (Pou2f3) | 4.84 | 0.04 | | -6.91 | 8.09E-36 |
| Trans-acting transcription factor 6 (Sp6) | 6.18 | 0.03 | | -7.68 | 4.02E-67 |
| Transcription factor AP-2, epsilon (Tfap2e) | 3.96 | 0.01 | | -8.63 | 1.07E-26 |
| Atonal bHLH transcription factor 1 (Atoh1) | 1.02 | 0.01 | | -6.67 | 6.61E-07 |

#Gene expression levels were measured using the FPKM method. FPKM, fragments per kilobase of transcript per million fragments mapped
