# Supplementary material for: Comprehensive RNA sequencing in primary murine keratinocytes and fibroblasts identifies novel biomarkers and provides potential therapeutic targets for skin-related diseases
Source: Cell Mol Biol Lett. 2021 Oct 3;26:42. doi: 10.1186/s11658-021-00285-6 (PMC8489068; doi:10.1186/s11658-021-00285-6)
Supplement: Supplementary file 7 — Additional file 7: Table S7. Gene expression levels of growth factor. [file 11658_2021_285_MOESM7_ESM.docx]

**Table S7.** Gene expression levels of growth factor

| Gene name | Gene Expression（FPKM**^#^**） | | log_2_ Fold Change (Fibroblast/Keratinocyte) | FDR |
| --- | --- | --- | --- | --- |
|  | Keratinocyte | Fibroblast |  |  |
| Connective tissue growth factor (Ctgf) | 468.88 | 218.51 | -1.10 | 0 |
| Insulin-like growth factor 2(Igf2) | 44.64 | 169.27 | 1.92 | 0 |
| Vascular endothelial growth factor D (Vegfd) | 3.46 | 61.45 | 4.15 | 2.81E-219 |
| Transforming growth factor, beta 3(Tgfb3) | 18.2 | 50.67 | 1.48 | 1.62E-121 |
| Vascular endothelial growth factor A (Vegfa) | 17.43 | 40.79 | 1.23 | 2.39E-70 |
| Transforming growth factor, beta induced (Tgfbi) | 60.47 | 23.11 | -1.39 | 1.30E-103 |
| Fibroblast growth factor 7(Fgf7) | 1.33 | 15.28 | 3.52 | 4.71E-81 |
| Vascular endothelial growth factor C (Vegfc) | 4.55 | 11.94 | 1.39 | 7.15E-15 |
| Nerve growth factor (Ngf) | 22.61 | 9.67 | -1.23 | 6.48E-14 |
| Heparin-binding EGF-like growth factor (Hbegf) | 20.34 | 5.54 | -1.88 | 2.63E-48 |
| Fibroblast growth factor 10(Fgf10) | 0.28 | 4.81 | 4.10 | 8.68E-51 |
| Fibroblast growth factor 2(Fgf2) | 0.54 | 4.21 | 2.96 | 7.11E-05 |
| Insulin-like growth factor 1(Igf1) | 0.78 | 3.36 | 2.11 | 7.30E-29 |
| Fibroblast growth factor 18(Fgf18) | 0.21 | 2.53 | 3.59 | 2.75E-08 |
| Platelet derived growth factor, alpha (Pdgfa) | 37.13 | 2.31 | -4.01 | 2.17E-120 |
| Fibroblast growth factor 23(Fgf23) | 0.04 | 2.19 | 5.77 | 1.99E-16 |
| Platelet-derived growth factor, D Polypeptide (Pdgfd) | 0.29 | 1.92 | 2.73 | 5.13E-06 |
| Placental growth factor (Pgf) | 3.85 | 1.5 | -1.36 | 0 |
| Fibroblast growth factor 5(Fgf5) | 0.12 | 1.26 | 3.39 | 8.54E-07 |
| Platelet derived growth factor, B Polypeptide (Pdgfb) | 60.38 | 1.12 | -5.75 | 0 |
| Hepatocyte growth factor (Hgf) | 0.04 | 0.97 | 4.60 | 1.27E-06 |
| Transforming growth factor alpha (Tgfa) | 12.77 | 0.39 | -5.03 | 4.56E-154 |

#Gene expression levels were measured using the FPKM method. FPKM, fragments per kilobase of transcript per million fragments mapped
