# Supplementary material for: Comprehensive RNA sequencing in primary murine keratinocytes and fibroblasts identifies novel biomarkers and provides potential therapeutic targets for skin-related diseases
Source: Cell Mol Biol Lett. 2021 Oct 3;26:42. doi: 10.1186/s11658-021-00285-6 (PMC8489068; doi:10.1186/s11658-021-00285-6)
Supplement: Supplementary file 8 — Additional file 8: Table S8. Primers sequences used for qRT-PCR to validate the RNA-Seq results. [file 11658_2021_285_MOESM8_ESM.docx]

**Table S8**. Primers sequences used for qRT-PCR to validate the RNA-Seq results

| Gene | Primer | Sequence |
| --- | --- | --- |
| Plac9a | Forward | TATGGAGGAGACGGTGGAGA |
|  | Reverse | CCTGTGGGAAGGTTTGAAGC |
| Ccl12 | Forward | CCCAGTCACGTGCTGTTAT |
|  | Reverse | GGCTGCTTGTGATTCTCCTG |
| Car3 | Forward | GACCTATCACGGCTCCTTCA |
|  | Reverse | GTCATGGGCTCTTTGAGCAG |
| Plac8 | Forward | GGCCATGAGGACTCTCTACC |
|  | Reverse | GTTCATGGCTCTCCTCCTGT |
| Agtr2 | Forward | TCCTCCTGGGATTCACCAAC |
|  | Reverse | GCCTTGGAGCCAAGTAATGG |
| Lce3c | Forward | TCTGGAGGAATCAGCATCCC |
|  | Reverse | TCCACAGGAAGGAAGGGAAC |
| Ccer2 | Forward | TGCTTCGTGTAACCACCTCT |
|  | Reverse | TCAATGGAGCTGAAGAGGCA |
| Cnfn | Forward | AACACCTGCTACCAGACACA |
|  | Reverse | CCAAAGTCATCGGAGATGCG |
| Sprr2h | Forward | CCCTGTACTTCAGCCTTCCA |
|  | Reverse | AGACTGCTGCAACTCTTCCT |
| Prnd | Forward | CCCTGTACTTCAGCCTTCCA |
|  | Reverse | AGACTGCTGCAACTCTTCCT |
| Col1a1 | Forward | GCTCCTCTTAGGGGCCACT |
|  | Reverse | ATTGGGGACCCTTAGGCCAT |
| Sparc | Forward | GTGGAAATGGGAGAATTTGAGGA |
|  | Reverse | CTCACACACCTTGCCATGTTT |
| Col13a1 | Forward | GGAGCACCTGGACTAGACG |
|  | Reverse | GCCTTGGACTGGTAAGCCAT |
| Dcn | Forward | TCTTGGGCTGGACCATTTGAA |
|  | Reverse | CATCGGTAGGGGCACATAGA |
| Cldn4 | Forward | GTCCTGGGAATCTCCTTGGC |
|  | Reverse | TCTGTGCCGTGACGATGTTG |
| Krt16 | Forward | GGTGGCCTCTAACAGTGATCT |
|  | Reverse | TGCATACAGTATCTGCCTTTGG |
| Sfn | Forward | GTGTGTGCGACACCGTACT |
|  | Reverse | CTCGGCTAGGTAGCGGTAG |
| Gapdh | Forward | GCACAGTCAAGGCCGAGAAT |
|  | Reverse | GCCTTCTCCATGGTGGTGAA |
