# Supplementary figures and images for: Comprehensive RNA sequencing in primary murine keratinocytes and fibroblasts identifies novel biomarkers and provides potential therapeutic targets for skin-related diseases
Source: Cell Mol Biol Lett. 2021 Oct 3;26:42. doi: 10.1186/s11658-021-00285-6 (PMC8489068; doi:10.1186/s11658-021-00285-6)

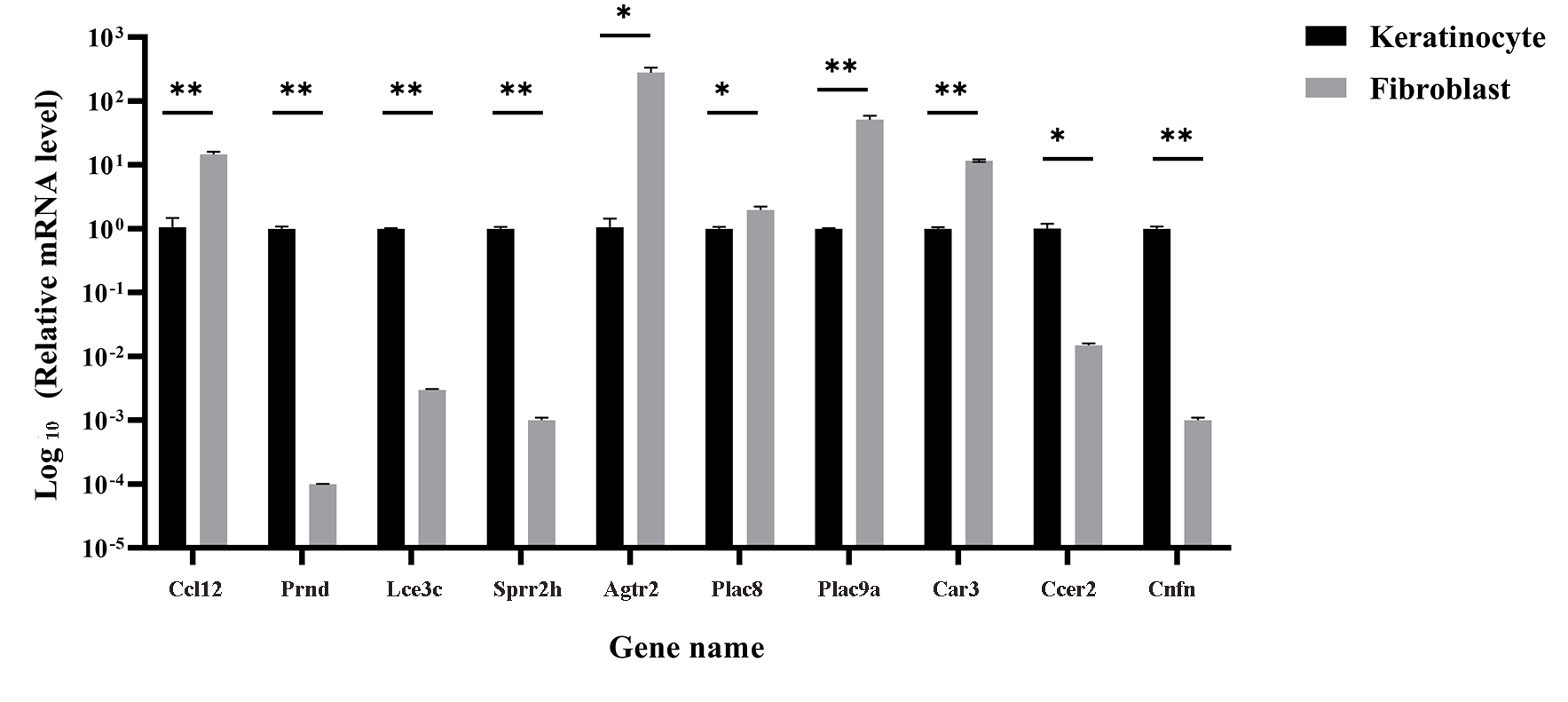

Supplement: Supplementary file 9 — Additional file 9: Figure S1. Validation of RNA-Seq data using qRT-PCR. Ten genes were randomly selected to be analyzed by qRT-PCR. The relative mRNA expression was expressed as 2−ΔΔCT. The Gapdh gene served as an internal reference to normalize the target gene expression. Mean ± SD (n = 3). *P < 0.05, **P < 0.01 [file 11658_2021_285_MOESM9_ESM.tif]
